# Integrative Genomic and Transcriptomic Analyses of Tumor Suppressor Genes and Their Role on Tumor Microenvironment and Immunity in Lung Squamous Cell Carcinoma

**DOI:** 10.3389/fimmu.2021.598671

**Published:** 2021-02-25

**Authors:** Ahreum Kim, Sun Min Lim, Joo-Hang Kim, Jeong-Sun Seo

**Affiliations:** ^1^ Department of Medicine, CHA University School of Medicine, Seongnam, South Korea; ^2^ Precision Medicine Center, Seoul National University Bundang Hospital, Seongnamsi, South Korea; ^3^ Division of Medical Oncology, Department of Internal Medicine, Yonsei Cancer Center, Yonsei University College of Medicine, Seoul, South Korea; ^4^ Department of Internal Medicine, CHA Bundang Medical Center, CHA University, Seongnam-si, South Korea; ^5^ Precision Medicine Institute, Macrogen Inc., Seoul, South Korea

**Keywords:** tumor suppressor gene, tumor microenvironment, The Cancer Genome Atlas, lung squamous cell carcinoma, lung adenocarcinoma

## Abstract

Non-small-cell lung cancers (NSCLCs) are largely classified into lung adenocarcinoma (LUAD) and lung squamous cell carcinoma (LUSC), which have different therapeutic options according to its molecular profiles and immune checkpoint expression, especially PD-L1, which is a suppressive factor in the tumor microenvironment. The tumor microenvironment can be altered by the genomic mutations on specific innate immune genes as well as tumor suppressor genes, so it is essential to comprehend the association between tumor microenvironment and tumor suppressor genes to discover the promising immunotherapeutic strategy to overcome the resistance of immune check point blockade. In this study, we aimed to analyze how the somatic mutations in tumor suppressor genes affect the tumor immune microenvironment through a comprehensive analysis of mutational profiling on the representative tumor suppressor genes (*TP53, CDKN2A, PTEN, RB1, BRCA1*, *BRCA2*) and immune gene expression in The Cancer Genome Atlas (TCGA) 155 lung squamous cell carcinoma (LUSC) and 196 lung adenocarcinoma (LUAD) samples. Several microenvironmental factors, such as the infiltrating immune and stromal cells, were suppressed by the mutated tumor suppressor genes in LUSC, unlike in the LUAD samples. In particular, infiltrating immune cells such as macrophage, neutrophil, and dendritic cells were significantly reduced in tumors with mutated tumor suppressor genes’ group. In addition, the gene expressions for interleukin production and lymphocyte differentiation and *PGC, C7, HGF, PLA2G2A, IL1RL1, CCR2, ALOX15B, CXCL11, FCN3* were significantly down-regulated, which were key immune genes for the cross-talk between LUSC microenvironment and tumor suppressors. Therefore, we generated evidence that TSG mutations in LUSC have an impact on tumor immune microenvironment, which suggests that TSG non-mutated patients will have the more inflamed tumors and are more likely to respond to immune checkpoint blockade therapy.

## Introduction

Lung cancer is one of the most common cancers and the cause of more than 25% of all cancer related deaths in men and women around the world (1, 2). Non-small-cell lung cancers (NSCLCs) consist of approximately 80% of lung cancers, which is classified into lung adenocarcinoma (LUAD) and lung squamous cell carcinoma (LUSC), and they have distinct molecular profiles and therapeutic options according to the genotypes (3). Especially, epidermal growth factor mutation (EGFR) tyrosine kinase inhibitor, which is widely used for the targeted therapy option and combination therapy with chemotherapy, has emerged as a promising treatment in LUAD patients, whereas LUSC has no targetable treatments for predisposed specific genetic alterations owing to the largely unknown underlying molecular mechanisms of LUSC pathogenesis (4–7).

The progression of cancer can be altered by the activity of specific innate immune cells, which have a significant association with the presence of tumor suppressors (8). The tumor-associated macrophages (TAMs) and regulatory B cells have proven to have a strong association with cancer progression and metastasis, and the mutations in the tumor suppressor genes could regulate the function of immune cells, particularly macrophage in preinvasive lesions (9–11). Additionally, the macrophages, which were differentiated from immature monocytes by cytokine, could enhance tumor suppressor activity *via* NF-kB pathways (12). The tumor mutation burden (TMB) and neoantigen burden are closely correlated with immune response including cytolytic activity and immune checkpoints (13, 14). Moreover, recent studies suggest that immunogenic gene expression is correlated with the response to therapy (15, 16).

The genomic alterations on tumor suppressor genes as well as mutational burden considering the immune gene expression profiles could provide the potential consequence of genomic alterations in TSG on the tumor microenvironment (TME) and immune checkpoints (17–19). Understanding the link between TME and tumor suppressors is indispensable to find the immune interaction between infiltrating immune and stromal cells and explore strategies to optimize immune checkpoint blockade therapy in NSCLC.

Therefore, we focused on the role of tumor suppressors on the immunity in TME to understand the fundamental mechanism of tumor suppressors in cancer immunity and predict the patient’s response to immunotherapy.

## Materials and Methods

### Samples and Pre-Processing

We used whole exome and transcriptome data of TCGA 155 LUSC and 196 LUAD samples, respectively. The 101 Korean LUSC cohort data which was previously published (20), was used for the validation of the study. IRB review for use of the TCGA patients data was exempted under TCGA policies which were in accordance with Common Rule. Also, the 101 Korean LUSC cohort was previously approved by the Institutional Review Board of Seoul National University Hospital

The data preprocessing was performed by the previously reported pipeline (20–22). The number of raw reads (HTSeq count for Ensembl annotated gene) was transformed to variance transformed data (R package ‘DESeq2’) for transcriptomic sequencing analysis. The HTseq count values were converted to Fragments Per Kilobase Million (FPKM) by R package ‘edgeR’. Also, the annotated VCF mutation files through the Genomic Data Commons Data Portal (GDC Data Portal) were assessed and downloaded for genomic analysis.

### Classification of TSG Subtype and Mutational Profiling

The subtypes considering tumor suppressor genes (TSGs) were classified based on the presence and number of the non-synonymous somatic mutations (missense, nonsense, frame shift insertion, frame shift deletion, In-frame insertion, In-frame deletion, and splice site mutation) in several key TSGs (*TP53, CDKN2A, PTEN, RB1, BRCA1, BRCA2*), which were previously reported as the representative TSGs (23).

Considering the presence of mutated TSGs, we defined TSG non-mutated group as patients having no mutated TSGs, and TSG mutated group as patients having equal to or greater than one mutated TSGs. In addition, four subgroups were further defined as non-TSG group (no mutated TSGs), TSG-1 (one TSG mutation), TSG-2 (two TSG mutations), and TSG-3 (equal to or greater than three TSG mutations) by considering the number of the mutations on TSGs.

The mutation profiling on representative TSGs was categorized and visualized with the mutation types and frequency. The number of non-synonymous somatic mutations for each patient was computed and compared between defined subgroups across 155 TCGA LUSC samples.

### Analysis of Differentially Expressed Genes

The up- or down-regulated differentially expressed genes (DEGs) of TSG mutated group compared to TSG non-mutated group and the adjusted p value were estimated by previously reported method and significance criteria (adjusted *P*  <  0.05, |Log2 (fold change)| ≥ 1, and base mean ≥ 100)(R package ‘DESeq’) (21). The gene enrichment analysis was performed by using the computed DEGs in the TSG loss subtype *via* Gene Ontology (GO) gene sets in the Molecular Signatures Database (MSigDB) (GSEA web version).

### Estimation of Infiltrating Immune Cells and Immune Activity

Several immune factors such as the infiltrating immune cells (B cells, CD4+ T cells, CD8+ T cells, neutrophil, macrophages, and dendritic cells) and immune activity (stromal, immune, cytolytic score, and tumor purity) were predicted by Tumor IMune Estimation Resource (TIMER) and Estimation of STromal and Immune cells in MAlignant Tumors using Expression data (ESTIMATE) algorithm (24, 25). The computed values for immune factors were statistically analyzed between subtypes considering the distribution and number of samples in each group.

### Calculation of Gene Expression Level of Immune Genes

To represent the gene expression value, the variance stabilized transformations (VST) normalized expression level were converted from raw read counts (HTseq) by R package ‘DESeq2’, and the VST normalized expression of the selected immune genes in TSG subtypes was box-plotted with the corresponding Mann–Whitney U or unpaired Student t-test depending on the sample distribution with Shapiro–Wilk normality test (R package ‘ggplot2’) (26).

### Characterization of Tumor Microenvironment and Clinical Association in TSG Loss Group

The clinical features such as TSG subtypes, gender, race, age, stage, smoking status, site of resection, location in lung parenchyma, new tumor event after initial treatment, person neoplasm cancer status, pT, pN, pM, dimension of sample/specimen (longest, shortest, and intermediate) were additionally summarized (**Supplementary Table 1**). The association of TSG subtypes with the immune checkpoint genes, clinical outcome, and parameters was analyzed and visualized with the statistical analysis and R package ‘ggplot2’. The expression (FPKM) of signature immune genes was adjusted to median-centered and log2 transformed by R package ‘edge R’ and Cluster 3.0.

Using the 144 TCGA LUSC samples who were provided with all clinical information, overall survival rate and hazardous ratio in TSG subtypes were computed by Kaplan–Meier estimates and a log-rank test (R packages ‘survival’ and ‘surviminer’) from the obtained TCGA clinical file (TMN stage, survival, age, sex, smoking status, location in lung parenchyma, dimension of sample/specimen, new tumor event after initial treatment), mutation load, and identified immune genes such as *KLK5, PGC, C7, HGF, PLA2G2A, IL1RL1, CCR2, ALOX15B, CXCL11*, and *FCN3*. Univariate and multivariable Cox regression models for overall survival were estimated by using the R package ‘surviminer’ in LUSC patients.

## Results

### Identification of TSG Subtypes in TCGA LUSC

**Figure 1:**
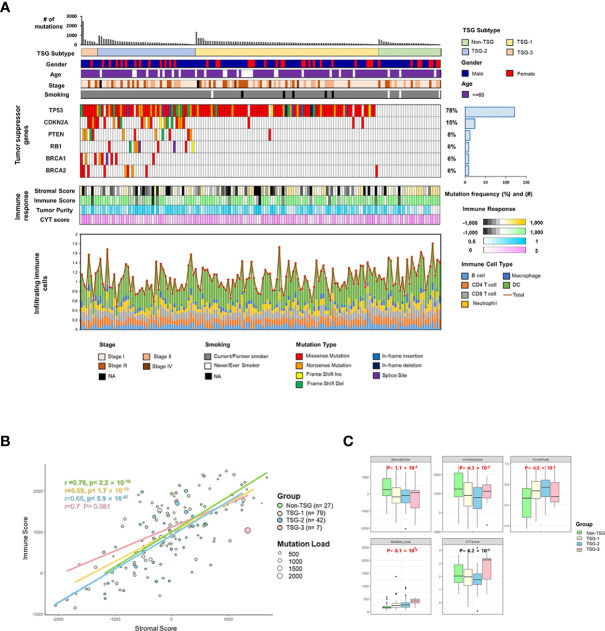
Identification of TSG subtypes in TCGA LUSC (n = 155). **(A)** The distribution of somatic nonsynonymous mutations (missense, nonsense, frame shift insertion, frame shift deletion, In-frame insertion, In-frame deletion, and splice site mutation) and TSG subtypes according to the number of mutations in TSGs were described across 155 TCGA LUSC samples, and the mutations on tumor suppressor genes were categorized with the mutation types and frequency. The four different indicators for the immune response (stromal and immune score, tumor purity, and cytolytic score) and infiltrating immune cells (B cells, CD4^+^ T cells, CD8^+^ T cells, neutrophil, macrophages, and dendritic cells) across the samples were displayed in each column. **(B)** The correlation between immune score and stromal score along with the mutation burden was plotted in each of the four TSG subtypes with Pearson’s correlation coefficient. **(C)** The immune factors (immune and stromal scores, tumor purity, mutation load, and cytolytic score) were box-plotted in four TSG subtypes. Each p-value was indicated by each of the subtypes (Kruskal–Wallis or one-way ANOVA test).

In this study, the distribution of somatic nonsynonymous mutations and the classification of TSG subtypes according to the number of mutations in TSG were described across 155 TCGA LUSC samples, and the somatic mutations located on tumor suppressor genes were categorized with its mutational types and frequency (**Figure 1**).

The TSG subtypes were defined by using the presence of the mutations in the six different tumor suppressor genes such as *TP53, CDKN2A, PTEN, RB1, BRCA1*, and *BRCA2*, which were previously reported as the representative TSGs (23). Two different subgroup classifications were applied to the LUSC samples according to the presence and the number of mutated TSGs.

Among the mutations in TSGs, the mutations encoding *TP53* had the highest mutation frequency (78%), followed by *CDKN2A* (15%), *PTEN* (8%), *BRCA1* (6%), *BRCA2* (6%) in the order of frequency. Each TSG mutated and TSG non-mutated groups had different patterns in four indicators for the immune response [stromal score, immune score, tumor purity, and cytolytic activity score (CYT score)], which CYT score represented the degree of cell destruction by immune cells (27).

In order to identify the impact of TMB on the tumor microenvironment, the correlation between immune and stromal scores along with the TMB values was plotted according to the number of mutated TSGs, and each immunogenic and microenvironmental factor was compared in each four TSG subtypes (**Figures 1B, C**). The tumor microenvironment factors as represented by immune and stromal score, tumor purity as well as mutation burdens were statistically different between subtypes. However, there was no significant difference in correlations between stromal and immune scores among the four TSG subgroups (non-TSG: Pearson’s *r*  =  0.78; TSG-1 Pearson’s *r*  =  0.69; TSG-2: Pearson’s *r*  =  0.68; TSG-3: Pearson’s *r*  =  0.7).

In the case of the TSG non-mutated and TSG mutated subtypes according to the presence of mutated TGS, the TSG mutated subgroup, which had equal to or greater than one mutation in TSGs (n = 128), had lower stromal and immune score than the TSG non-mutated subgroup (n = 27) which also had higher tumor purity. Also, the overall infiltrating immune cells [CD8^+^ T cells, CD4^+^ T cells, macrophage, dendritic cells (DCs), and B cells, neutrophils] were more abundant in the TSG non-mutated subgroup. In addition, the correlation between the number of mutations in the TSG and the immune score was investigated, and there was a negative association between them (Spearman’s correlation r = −0.18, *P* < 0.05) (**Supplementary Figure 1**).

### The Association Between Tumor Microenvironment and Immune Response

The different patterns of immune response in LUAD and LUSC according to the presence of TSG mutation were identified, and all factors representing immune response (stromal and immune score, ESTIMATE score, tumor purity, CYT score, B cells, CD4^+^ T cells, CD8^+^ T cells, neutrophils, macrophages, and dendritic cells) had no significant difference between TSG mutated and TSG non-mutated groups in LUAD whereas the majority of immune factors such as stromal and immune score, and ESTIMATE score as well as infiltrating immune cells (macrophage, dendritic cells, and neutrophils) were significantly lower in the TSG mutated group in LUSC (**Supplementary Figure 2**).

**Figure 2:**
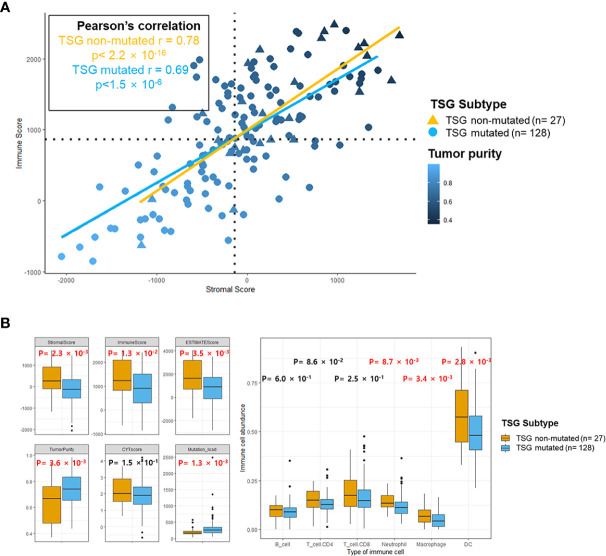
The immune landscape of the microenvironment in TSG subtypes. **(A)** Scatterplots between stromal and immune scores with tumor purity gradient were shown, and its correlation coefficient was indicated by each of the subtypes. The color grading corresponds to the tumor purity, indexed as shown on the color bar at the bottom right of the panel. The median scores for stromal and immune scores were indicated by dashed lines under the horizontal (x) and vertical (y) axis. **(B)** Several indicators for immune response and abundance of infiltrating B cells, CD4^+^ T cells, CD8^+^ T cells, neutrophil, macrophages, and dendritic cells in two subtypes were estimated, and each p-value was indicated by each of the subtypes (Mann–Whitney U test and unpaired t-test). Box represents the median (thick line) and the quartiles (line).

In order to analyze the impact of TSG mutations on the TME of LUSC, the association between infiltrating immune cells and TME factors was investigated in each TSG subtype. The correlation coefficient (r) between stromal and immune scores was 0.78 in the TSG non-mutated group and 0.69 in the TSG mutated group, and the tumor purity was lower as the stromal and immune scores were increasing in both groups (**Figure 2A**), and this revealed that the stromal scores of the TSG non-mutated group were more positively associated with the immune scores.

Especially, the immune factors for presenting TME (stromal score, immune score, estimate score, and tumor purity) were significantly lower in the TSG mutated group than in the TSG non-mutated group (**Figure 2B**). Also, the infiltrating immune cells such as neutrophils, macrophage, dendritic cells were significantly increased in the TSG non-mutated group, and these results accorded closely with the result of previous LUSC cohorts (**Supplementary Figure 3**).

### Gene Enrichment Analysis in TSG Subtypes

**Figure 3:**
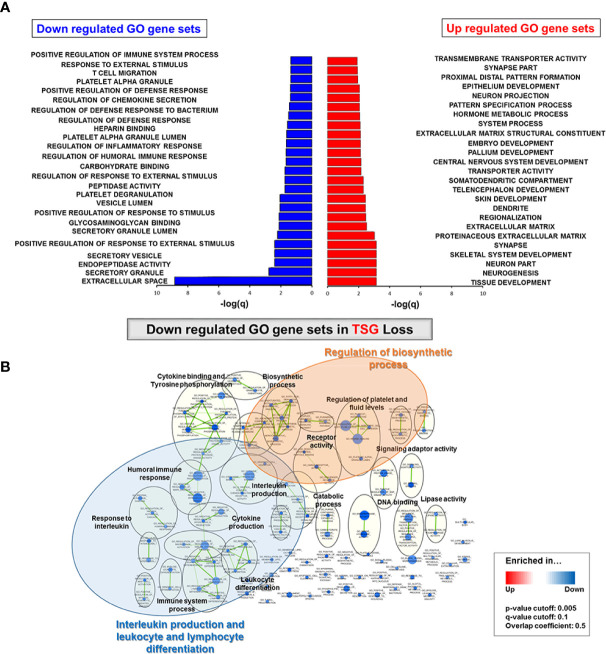
Gene enrichment analysis in TSG subtypes. **(A)** Top 25 GO gene sets in either down- and up-regulated GO gene sets were determined based on the rank of enrichment –log_10_(q value) of the pathway and the matched significance criteria (P-value < 0.05 and FDR q value < 0.1) **(B)** Network visualization based on gene enrichment analysis. Blue nodes represent the down-regulated gene sets in TSG subtype. Genes in significant networks were annotated and grouped with simplified GO terms. Networks meeting the cut-off conditions detailed at the bottom of the figure (right) were visualized with the Enrichment Map plugin for Cytoscape (P ≤ 0.05, FDR q value ≤ 0.01, and similarity ≤ 0.5).

The top 25 Gene Ontology (GO) gene sets in either down- and up-regulated gene sets were investigated in the TSG mutated group compared to the TSG non-mutated group based on the rank of enrichment –log_10_(q value) of the pathway and the matched significance criteria (*P*-value < 0.05 and FDR q value < 0.1) (**Figure 3A**). Through the enrichment analysis, the immune related gene sets such as regulation of humoral immune response, regulation of inflammatory response, regulation of chemokine secretion, and T cell migration were significantly down-regulated whereas the tissue development, neurogenesis, and neuron part gene sets were significantly up-regulated in the TSG mutated group.

When we conducted the network visualization on gene enrichment analysis, the gene sets for interleukin production and leukocyte and lymphocyte differentiation were down-regulated in the TSG mutated group as well as the regulation of biosynthetic process (*P* ≤ 0.05, FDR q value ≤ 0.01, and similarity ≤ 0.5) (**Figure 3B**). Next, we visualized each network of down-regulated GO gene sets in *TP53* and *CDKN2A* mutated group *via* gene enrichment analysis. Similarly, the interleukin production, leukocyte and lymphocyte differentiation, and mediated immunity were significantly down-regulated in both *TP53* and *CDKN2A-*mutated groups (**Supplementary Figure 4**).

### Impacts of TSG Loss on TME and Immune Response in LUSC

**Figure 4:**
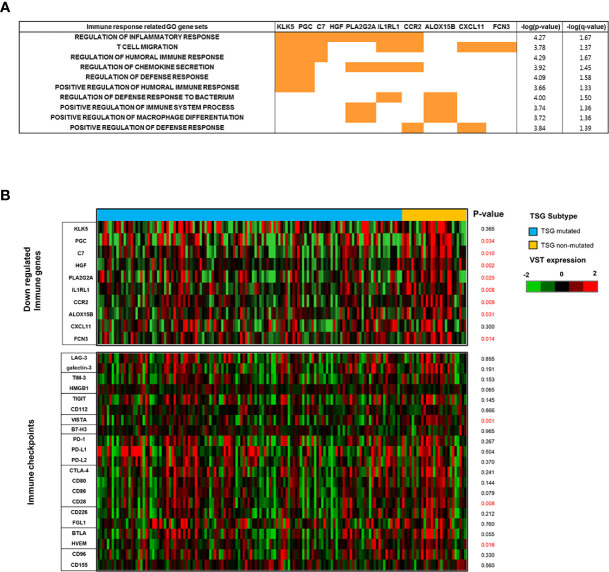
Impacts of mutated TSGs on immune response in LUSC. **(A)** The down-regulated immune genes in TSG subtype were classified by each enriched GO immune gene sets and indicated with the p and q values. **(B)** The expression of the selected immune genes was depicted in the heatmap with the computed p-value between TSG subtypes. The expression of immune checkpoint genes was analyzed and indicated with the p-value between TSG subtypes. All p-values were computed by Mann–Whitney U test or unpaired t-test based on the sample distribution.

The down-regulated immune genes in the TSG mutated group were classified by each enriched GO immune gene sets and indicated with the p and q values (**Figure 4A**). Statistically, *KLK5, PGC, C7, HGF, PLA2G2A, IL1RL1, CCR2, ALOX15B, CXCL11*, and *FCN3* were selected for the immune genes which could affect the TSG activity, and those immune gene expressions except for *KLK5* and *CXCL11* were significantly down-regulated in the TSG mutated group compared to the TSG non-mutated group (**Figure 4B** **and** **Supplementary Figure 5**). Among the emerging immune checkpoint receptors and their respective ligands, the expression of immune checkpoints such as VISTA and CD28 and ligands on APCs such HVEM were significantly up-regulated in the TSG mutated group, whereas other immune checkpoint molecules such as PD-1 and PD-L1 were not affected (28).

### The Association of TSG Subtype and the Clinical Outcome and Parameters

Kaplan–Meier survival analysis for 144 TCGA LUSC patients indicated that there was no significant difference in overall survival between the TSG non-mutated and TSG mutated subtypes as well as four TSG subtypes (Kaplan–Meier estimates; P = 0.11 for two TSG subtypes; P = 0.23 for four TSG subtypes, **Supplementary Figure 6**). In fact, subgroup classification was not a significant factor in determining the clinical association in LUSC samples. Also, the hazardous ratio along with the TSG subtypes, gender, age, stage, smoking status, TMB, pT, pN, pM, dimension of sample/specimen (longest, shortest, and intermediate), location in lung parenchyma, and selected immune gene expressions was analyzed, and several parameters such as *PLA2G2A, FCN3*, and new tumor event after initial treatment in these clinical factors had some prognostic value with univariate analysis (*PLA2G2A:* HR = 1.6, P = 0.046, *FCN3*: HR = 1.8, P = 0.024, and new tumor event after initial treatment: HR = 1.8, p = 0.034, **Table 1**). Also, it was confirmed that *PLA2G2A* and new tumor event after initial treatment were statistically significant prognostic value with multivariable analysis (*PLA2G2A:* HR = 1.8, p = 0.04 and new tumor event after initial treatment: HR = 2.1, p = 0.01).

**Table 1:** Cox proportional hazards model analysis of overall survival in LUSC patients.

Overall survival (n = 144)						
Variables	Univariate analysis			Multivariate analysis		
	HR	95% CI for HR	P -value	HR	95% CI for HR	P -value
**Age (≥60/<60)**	1.1	(0.57–2.1)	0.81			
**Sex (male/female)**	1.4	(0.80–2.5)	0.23			
**Smoking (smoker/non-smoker)**	0.3	(0.082–1.4))	0.20			
**pStage (II, III or IV/I)**	1.0	(1.0-1.0)	0.58			
**pN (I, II, III or X/0)**	1.0	(0.61–1.7)	0.92			
**pM (I or X/0)**	1.2	(0.37–3.8)	0.79			
**pT (II, III or IV/I)**	1.2	(0.71–2.2)	0.50			
**Location (peripheral/central)**	1.3	(0.52–3.2)	0.57			
**Longest dimension** **(≥median/<median)**	1.0	(0.60–1.6)	0.92			
**Intermediate dimension** **(≥median/<median)**	0.77	(0.47–1.2)	0.29			
**Shortest dimension** **(≥median/<median)**	0.77	(0.45–1.3)	0.35			
**New tumor event after initial treatment (YES/NO)**	1.8	(1.0–3.0)	0.034*	2.1	(1.2–3.6)	0.01**
**Mutation load (high/low)**	0.77	(0.48–1.2)	0.28			
**Group (TSG mutated group/TSG non-mutated group)**	0.64	(0.37–1.1)	0.11			
**KLK5 (high/low)**	1.0	(0.63–1.7)	0.92			
**PGC (high/low)**	1.2	(0.72–1.9)	0.55			
**C7(high/low)**	1.2	(0.75–2)	0.43			
**HGF(high/low)**	1.0	(0.65–1.7)	0.84			
**PLA2G2A (high/low)**	1.6	(1.0–2.7)	0.046*	1.8	(1.0–3.2)	0.04*
**IL1RL1 (high/low)**	0.75	(0.46–1.2)	0.23			
**CCR2(high/low)**	0.77	(0.48–1.2)	0.29			
**ALOX15B (high/low)**	1.2	(0.73–1.9)	0.50			
**CXCL11 (high/low)**	0.84	(0.52–.14)	0.49			
**FCN3(high/low)**	1.8	(1.1–2.9)	0.024*	1.5	(1.9–2.7)	0.12

HR, hazard ratio; CI, confidence interval. *p < 0.05; **p < 0.01.

## Discussion

The predictive and prognostic biomarkers for immune checkpoints inhibitors (ICIs) have been developed by estimating the PD-L1 expression level as well as the mutational burden in the TME, but these were not sufficient to predict response to the efficacy of immune checkpoint inhibitors, and it has not considered the relative contribution of each immune cells in the anti-tumor response (29–31). The cancer cells have heterogeneous PD-L1 expression, and the small biopsies and microarray could not detect the entire PD-L1 expression, which finally could give rise to the inconsistency with the surgically resected tissue samples (32). Also, previous studies demonstrated that the tumor suppressor inactivation enhanced inflammation and altered TME (33).

In our study, the genomic and transcriptomic analyses were useful in determining the impact of TSG loss on the TME and immunity in LUSC.

The correlation between immune and stromal scores along with the TMB values among four TSG subgroups indicated that the stromal and immune cells were strongly correlated with the mutational burden regardless of subtype, which was consistent with previous results that the tumor cells with high TMB and the formation of neoantigens had a decisive effect on the defective immune system and TME including tumor immunogenicity (22, 34).

Also, the negative association between the number of mutations in TSG and the immune score confirmed that the dysfunction in tumor suppressor genes such as p53 could have an impact on stromal and immune cells in the TME, which may accelerate tumor immune evasion, as described previously (35). The TME of LUSC considering mutational profiling was more useful in classifying TSG mutation mediated immune-deficient subtype and establishing personalized immunotherapy treatment options in LUSC patients (36, 37).

The abundant infiltrating immune cells such as neutrophils, macrophage, dendritic cells in the TSG non-mutated group demonstrated that the abundance of infiltrating immune cells was decreased by inactivation of tumor suppressor genes, which could lead to result in the malignant transformation in cancer cell (38). The infiltrating neutrophils in inflammatory environments could attract the macrophages and dendritic cells for enhancing tumor cell invasion and metastasis (39).

The interleukin production and lymphocyte differentiation were significantly down-regulated in the TSG mutated LUSC group. This suggests that several TSGs such as *RB1, TP53*, and *CDKN2A* in LUSC, which are involved in various cellular processes, including signaling pathways and DNA damage repairing system, could have disturbed the interleukin-5 pathway, PD-1 signaling, cytotoxic T lymphocyte pathway (40).

Previously, it was confirmed that the overexpression of interleukin 12 (*IL12*) induced the tumor-suppressive effect by mediating antitumor activity, and mutated tumor suppressor genes such as *TP53* potentially regulated the immune cell infiltration through its interaction with NF-kB and prevented activation of cytotoxic T cells, NK cells, and macrophages (38, 41, 42).

In addition, the newly identified *PGC, C7, HGF, PLA2G2A, IL1RL1, CCR2, ALOX15B, CXCL11*, and *FCN3* genes, which were down-regulated in the TSG mutated group, were key immune genes which might be involved in the crosstalk between cancer microenvironment and tumor suppressors. These detected immune response genes could be involved in regulating the immune cell proliferation upon TSG inactivation in cancer cells (18, 35, 40). Among the significantly up-regulated immune checkpoint expression in the TSG mutated group, VISTA, which is previously known as a PD-1 homolog and shares a sequence homology both to CD28 and B7 families, could be an immunotherapeutic target in the TSG non-mutated group by regulating the T cell function (43). Further validation of the function of key immune genes is needed to understand the impact on the TME of LUSC.

Unexpectedly, the number of mutations in TSGs had weak association with the survival, stage, and several clinical parameters similar to previous study (44). However, immune genes such as *PLA2G2A* and *FCN3* had a statistically significant association with overall survival, and the genomic alterations in TSGs had an impact on the reduction in interleukin production, leukocyte, and lymphocyte differentiation in TME, and understanding the interaction between TSG and TME will be useful to successfully treat LUSC patients.

Although we identified that TSG non-mutated patients may have more inflamed tumors, our study is limited in that we have not validated our findings in an independent patient cohort. We presume that patients without TSG mutation are more likely to respond to immune checkpoint blockade therapy, and this hypothesis needs to be further validated in a separate analysis involving LUSC patients who received anti-PD-1 or PD-L1 therapy. If these findings are proven, we may design a clinical trial using immune checkpoint inhibitor and designate TSG mutation status as a stratification factor or only include the patients without TSG mutation to improve the efficacy outcomes.

In conclusion, we showed that TSG mutations in LUSC may impact tumor immune microenvironment in that TSG non-mutated patients will have more inflamed tumors and are more likely to respond to immune checkpoint blockade therapy.

Establishing the role of tumor suppressor genes on the tumor immune environment could be useful to distinguish LUSC patients who may respond or not respond to immune checkpoint inhibitors if these findings are validated in prospective LUSC patient cohorts.

## Data Availability Statement

Publicly available datasets were analyzed in this study. This data can be found here: TCGA data portal (https://portal.gdc.cancer.gov/), NCBI Sequence Read Archive accessions (no. SRP114315).

## Author Contributions

J-SS and J-HK conceived and guided the whole research, and AK and SML designed the experiments. AK performed sequencing data processing, bioinformatics, and statistical analyses. AK and SML wrote the manuscript. All authors contributed to the article and approved the submitted version.

## Funding

This work has been supported by Macrogen Inc. (grant no. MGR17-05) and the National Research Foundation of Korea funded by the Korean government (2019R1A2C4069993).

## Conflict of Interest

J-SS was employed by the company Macrogen Inc.

The remaining authors declare that the research was conducted in the absence of any commercial or financial relationships that could be construed as a potentialconflict of interest.

The authors declare that this study received funding from Macrogen Inc. The funder had the following involvement in the study: the study design, the server for data processing, and the publication fee.
