# Supplementary material for: Integrative Genomic and Transcriptomic Analyses of Tumor Suppressor Genes and Their Role on Tumor Microenvironment and Immunity in Lung Squamous Cell Carcinoma
Source: Front Immunol. 2021 Feb 25;12:598671. doi: 10.3389/fimmu.2021.598671 (PMC7948518; doi:10.3389/fimmu.2021.598671)
Supplement: Supplementary file 1 [file DataSheet_1.pdf]

Supplementary Figures

Supplementary Fig. 1- Fig. 6

Supplementary Fig. 1 Correlation between the number of TSG mutations and immune score.

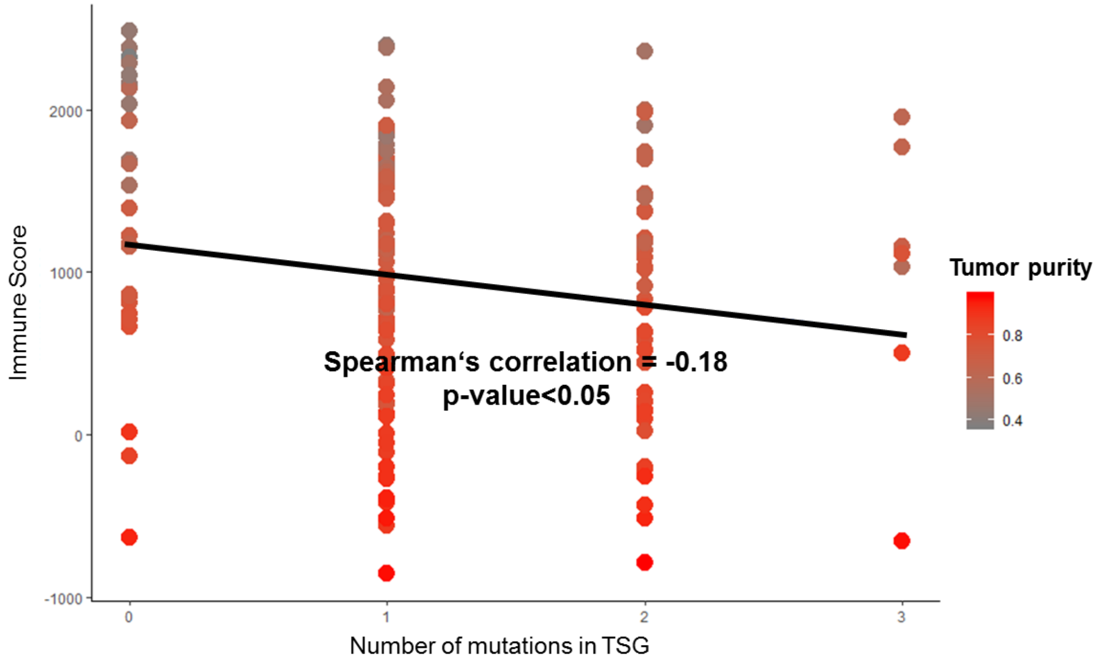

The correlation between the number of TSG mutations and immune score were plotted with Spearman's correlation coefficient.

Supplementary Fig.2 The immune landscape of the microenvironment in TSG LUAD and LSUC subtypes.

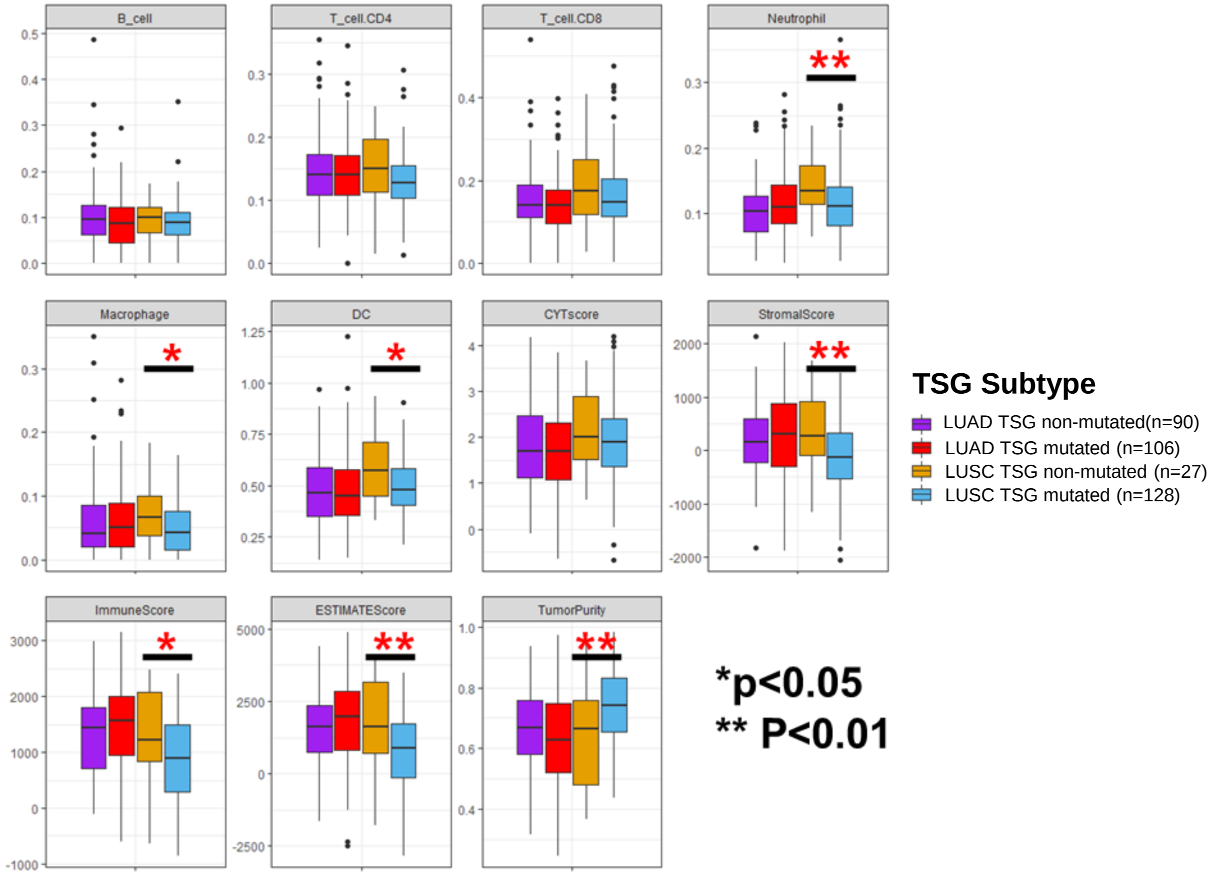

The different patterns of immune response in two TSG groups of LUAD and LUSC were estimated, and each p-value was indicated by each of the subtypes .

Supplementary Fig.3 Validation of immune response and infiltrating immune cells in two TSG groups

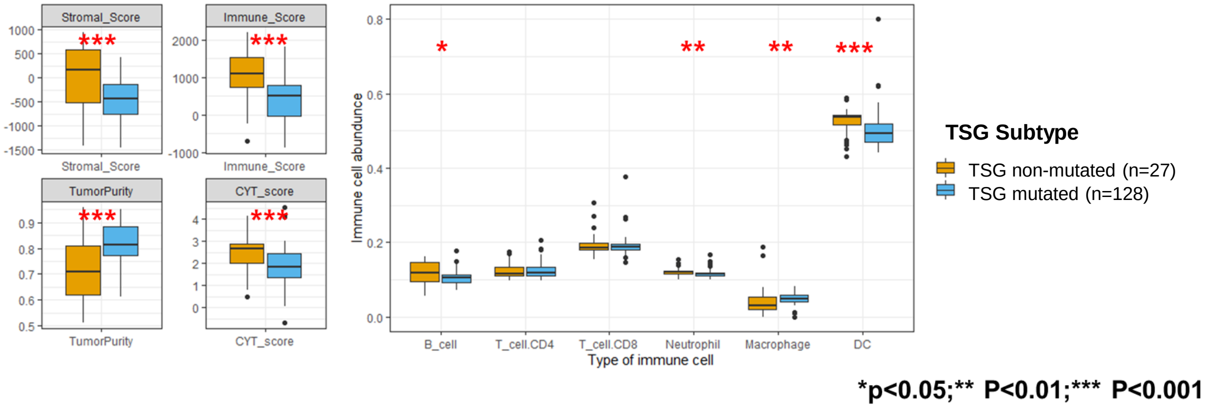

The different patterns of immune response and infiltrating immune cells in two TSG groups were validated by previous LUSC cohort.

Supplementary Fig.4 Gene enrichment analysis of down regulated GO gene sets in TP53 and CDKN2A mutated subtype.

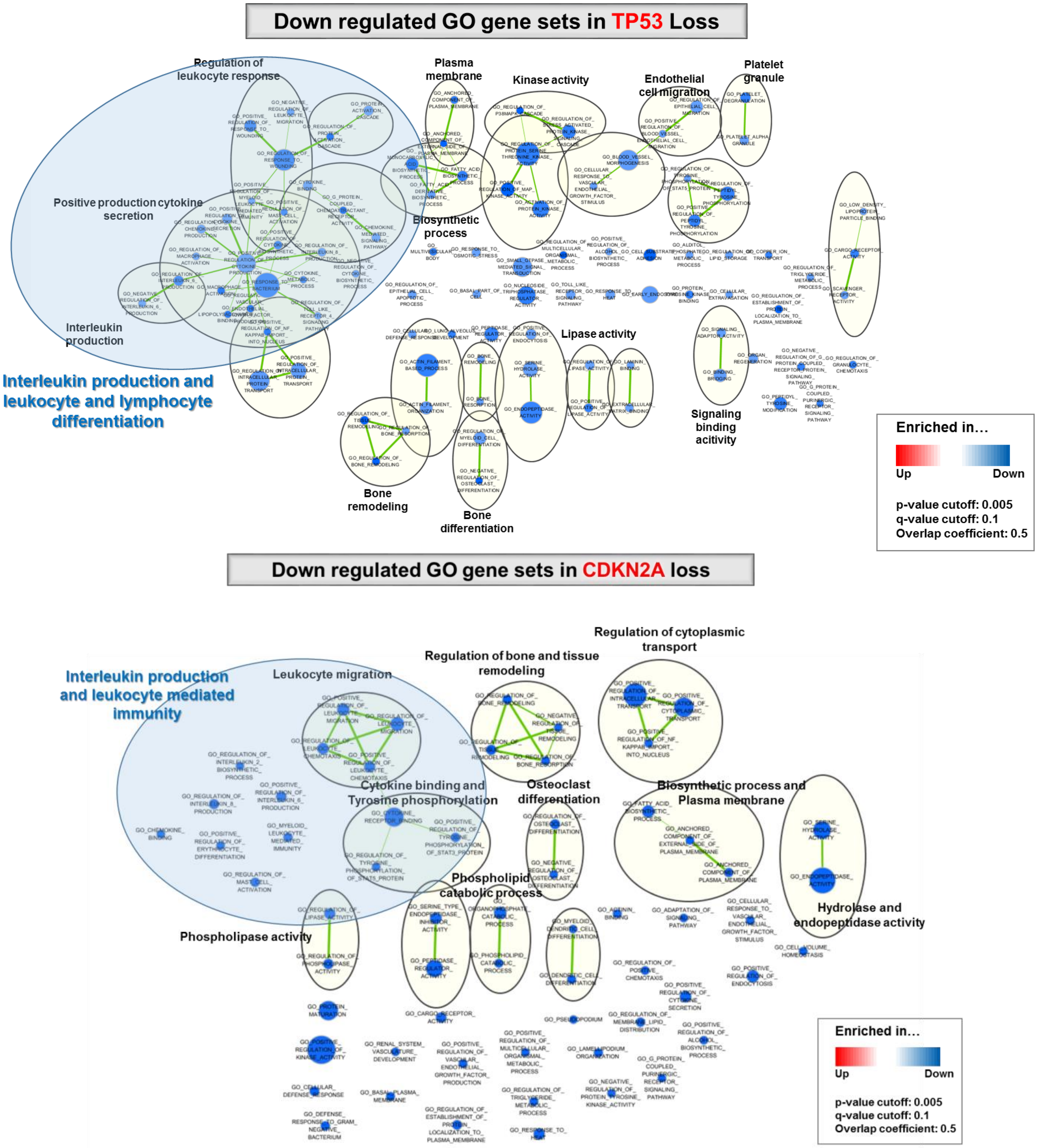

Network visualization based on gene enrichment analysis. Blue nodes represent the down regulated gene sets in TP53 loss and CDKN2A loss. Genes in significant networks were annotated and grouped with simplified GO terms

Supplementary Fig.5 The VST normalized expression level of the selected immune genes in TSG subtypes

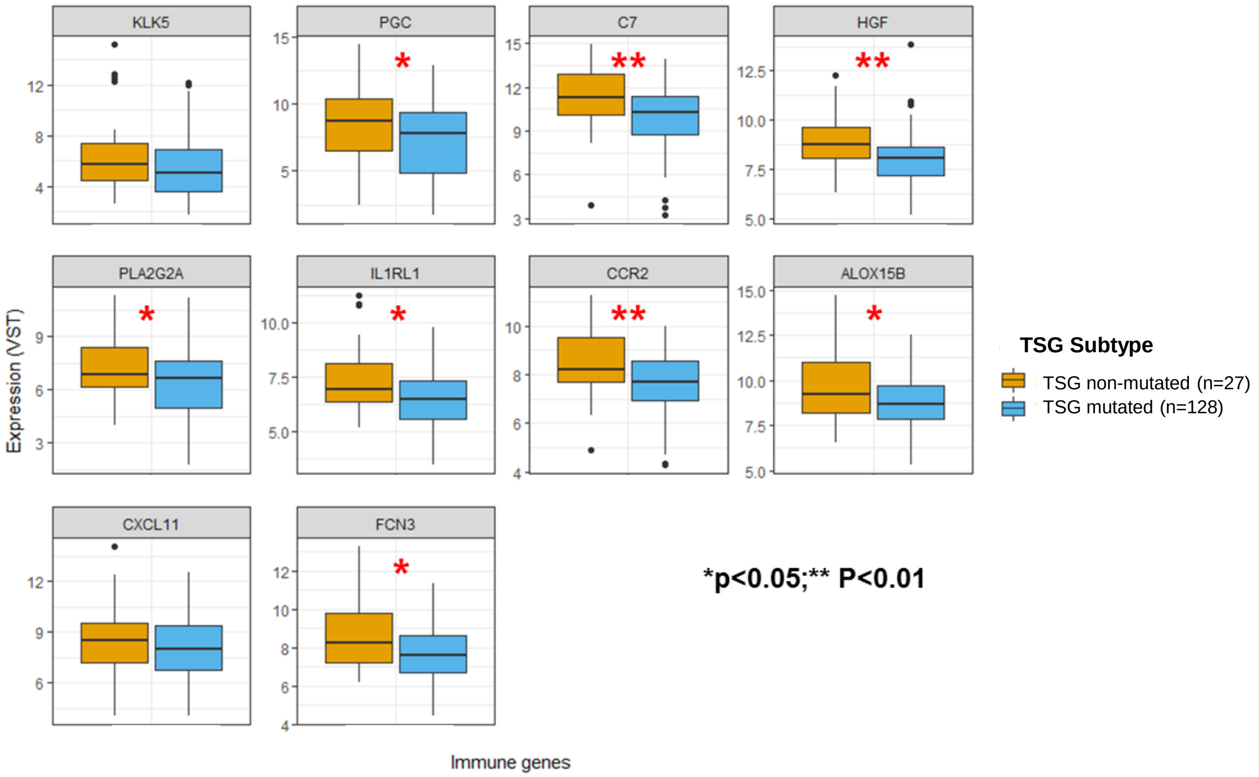

The VST normalized expression level of the selected immune genes in TSG subtypes was box-plotted with corresponding Mann-Whitney U or unpaired Student t test. Box represents the median (thick line) and the quartiles (line).

Supplementary Fig.6 The clinical association in TSG subtypes.

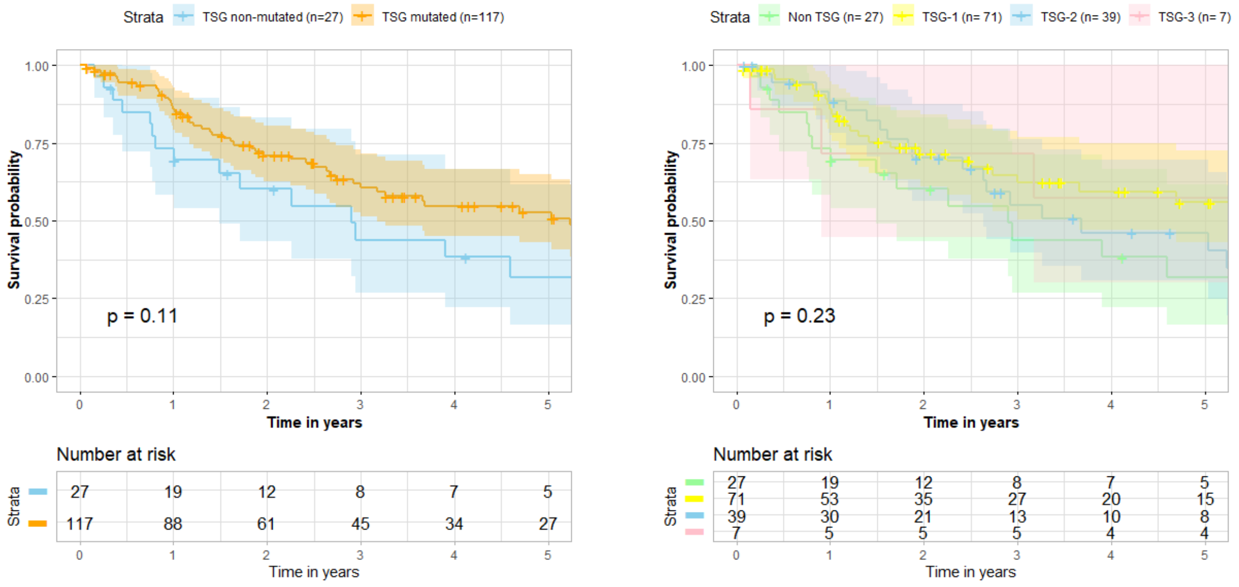

Survival curves for LUSC (n = 144) samples were visualized in two and four different TSG subtypes based on Kaplan-Meier estimates.
